# Nutritional quality of pre-packaged foods in Nigeria: findings from the NigeFE study

**DOI:** 10.3389/fnut.2026.1873374

**Published:** 2026-08-13

**Authors:** Gideon Onyedikachi Iheme, Barbara Adimchinobi Onyeonu, Favour Chiamaka Kalu, Uju Maryanne Onuorah, Favour Chinonyerem Ogu, Chinwe Adaugo Uzokwe, Chinyere Ikejiofor, Folake Olukemi Samuel

**Affiliations:** 1 Department of Food Studies, Nutrition and Dietetics, Uppsala University, Uppsala, Sweden; 2 Department of Human Nutrition and Dietetics, Michael Okpara University of Agriculture, Umudike, Nigeria; 3 Department of Nutrition and Dietetics, Federal University of Agriculture, Mubi, Nigeria; 4 Department of Nutrition and Dietetics, University of Nigeria Nsukka, Nsukka, Nigeria; 5 Department of Pharmacy and Health and Nutrition Sciences, University of Calabria, Arcavacata, Italy; 6 Nutritional Epidemiology Group, School of Food Science and Nutrition, University of Leeds, Leeds, United Kingdom; 7 Nutrition Unit, National Agency for Food and Drug Administration and Control, Lagos, Nigeria; 8 Department of Nutrition and Dietetics, University of Ibadan, Ibadan, Nigeria

**Keywords:** food labeling, NigeFE, nutrient profile, prepackaged foods, ultra-processed foods

## Abstract

**Background:**

The market penetration of pre-packaged foods, particularly less healthy and ultra-processed products such as instant noodles, sugar-sweetened beverages, snacks, and confectionery, has increased substantially in developing countries, including Nigeria. Growing consumption of these products has been associated with an increased burden of non-communicable diseases (NCDs), which account for approximately one-third of all deaths in Nigeria. Nevertheless, comprehensive evidence on their nutrient profiles remains scarce. Hence, our study aims to assess the nutritional quality of pre-packaged foods in Nigeria.

**Methods:**

This cross-sectional study was conducted to compile the nutritional information of 883 popular pre-packaged foods in the Nigerian market. These nutrients were profiled using the WHO Nutrient Profile Model for the African Region. Statistical analysis was done using IBM SPSS version 28.

**Results:**

Energy, fat, and protein contents were disclosed in majority (70.8–100%) of pre-packaged products, while the disclosure of cholesterol and sub-components of fat and sugar was considerably lower. Edible oil products contained the most energy (722.88 kcal), fat (84.5 g), saturated fat (27.2 g), monounsaturated fat (37.8 g), and polyunsaturated fat (28.5 g) values. Processed fish (54.17 g) and dairy (6.92 g) products had the highest cholesterol and trans-fat contents. Processed tuber (73.48 g), meat products (25 g), and snack food (6,448.78 mg) contained the highest carbohydrate, protein, and sodium, respectively. While a similar pattern was reported in the median values. However, notable differences between mean and median values were observed for selected nutrients and food categories, indicating skewed nutrient distributions.

**Conclusion:**

Marketing thresholds for one or more critical nutrients were exceeded by the majority of products, highlighting regulatory gaps and challenges in industry compliance. Stronger regulatory measures and support for improved nutrient disclosure and reformulation is needed to reduce the levels of these critical nutrients.

## 1 Introduction

The changing African food environment, a consequence of globalization, has intensified demand for convenience foods as a trade-off for the time and effort required for traditional food preparation (1). This shift, together with the relatively lower affordability of nutrient-dense fresh foods compared with many energy-dense packaged alternatives, has contributed to increased consumption of pre-packaged foods (2, 3). In parallel, the proliferation and aggressive marketing by the food industry have deepened penetration and consumer demand for pre-packaged food (4, 5). Pre-packaged foods have gradually become a mainstay in the African food system, including in Nigeria. However, public health concerns regarding the consumption of pre-packaged products are enormous, particularly ultra-processed foods, which represent the highest level of industrial processing in the NOVA classification system (6, 7). Beyond processing levels, most pre-packaged foods are also low in nutritional quality, containing high levels of saturated fats, trans-fatty acids, free sugars, or salt, and low levels of micronutrients (8). These unhealthy diets have been touted as the leading cause of non-communicable diseases, disability and death globally and in sub-Saharan Africa (8–10). Non-communicable diseases (NCDs) account for 75% of all non-pandemic-related deaths globally and 27% of all deaths in Nigeria, underscoring the urgent need to address diet-related and other modifiable risk factors (11, 12).

Addressing this requires a strong regulatory environment that is tailored to improving the healthiness of food environments. The World Health Organization (WHO) issued various recommendations to enhance the nutritional value and safety of processed food products, including limiting the intake of free sugars to less than 10% of total energy intake, with a further reduction to below 5% or approximately 25 grams (6 teaspoons) per day to provide additional health benefits (13). In furtherance of that, adults are recommended to consume less than 2,000 mg of sodium or 5 grams of salt and at least 3,510 mg of potassium per day, as high sodium intake or low potassium levels are associated with the increased risk of raised blood pressure, which in turn increases the risk of heart disease and stroke (14). Additionally, the WHO encourages implementing front-of-pack (FOP) labeling systems to help consumers make healthier choices (15). This is supported by the transition in national guidelines and regulations from voluntary to mandatory declaration of nutrients, regardless of claims statements, including nutrients of concern such as cholesterol, sodium, sugar, and trans-fat (16, 17). Accordingly, it is important to examine the extent to which these regulatory shifts are translated into actual implementation through the disclosure of these nutrients in pre-packaged foods, as well as whether the levels of key nutrients exceed recommended limits.

To achieve this, the World Health Organization suggested that countries develop nutrient profiling models to categorize foods based on their nutritional value (18). Nutrient profiling helps shape policies by demonstrating the distribution of food healthiness based on their nutrient composition. Different nutrient profiles (NPs) have been developed in Latin America to assess the nutritional quality of packaged food products. The Mexican NP was created as part of the warning-label regulation implemented in 2020, which includes five warning octagons (calories, sugar, sodium, saturated fats, and trans fats) and two warning rectangles (caffeine and non-nutritive sweeteners) (19). The Health Star Rating System in Australia and New Zealand, Nutri-Score in Europe, and the UK Food Standards Agency's Ofcom model are examples of popular Nutrient Profiling Models (20–22). Similar to other regions (23, 24). The WHO African Regional Nutrient Profile Model (NPM) serves as a regional template to guide national actors in developing locally relevant NPM (25), with significant implementation and adoption efforts recorded in Kenya (26) and Ghana (27). While consultations are underway to develop the Nutrient Profiling Model in Nigeria, the regional framework remains a valuable tool for profiling pre-packaged foods, thereby underscoring the urgency of a more committed approach to transforming the food environment.

Although evidence demonstrates that household expenditure and consumption of processed foods in Nigeria remain substantial, recent analyses indicate a slight decline in these trends over time (28–30). However, this apparent decrease may reflect differences in terminology, as “processed foods” in these studies often refer to baked goods or foods consumed outside the home, rather than specifically to pre-packaged products. In contrast, a recent national study found that nearly half of Nigerians consume less healthy foods, predominantly pre-packaged products such as instant noodles and soft drinks (31). Interestingly, pre-packaged foods have also been identified as major contributors to the consumption of fortified vegetable oil, sugar, flour, and salt in Nigeria (32). This indicates that while these products are often classified as unhealthy, their nutrient composition may either provide adequate amounts of essential nutrients or contain excessive levels of undesirable nutrients, underscoring the need for an in-depth investigation.

Therefore, as the body of evidence on nutrient profiling continues to expand globally, with studies emerging from countries such as China, Australia, New Zealand, and Kenya (33–35), recent studies reporting the nutrient composition of pre-packaged foods in Nigeria have been limited either by a narrow range of pre-packaged products examined (36) or by the focus on only select key nutrients (37). This study, therefore, provides a more comprehensive assessment of the nutritional quality of pre-packaged foods in Nigeria.

## 2 Methods

### 2.1 Study design

This descriptive cross-sectional content analysis is part of the preliminary data collection for the Food Label Analysis and Information Repository (FLAIR) database, Nigeria's repository for pre-packaged food and beverages. The FLAIR database is part of the broader NigeFE study, which generates evidence to monitor the food environment in Nigeria.

### 2.2 Data collection

Data were collected by trained enumerators who were nutrition and dietetics professionals with expertise in nutritional sciences and food labeling. Enumerator across participating states identified commonly available pre-packaged food products based on their knowledge of product availability in supermarkets and retail outlets, as well as their observations of consumer purchasing patterns within their local food environments. These inventories were subsequently compared across study locations, and products identified in multiple states were prioritized for inclusion to capture products with broad geographic availability and consumer reach. Following this process, a total of 883 pre-packaged food products with multi-location availability collected from supermarkets and retail stalls across major cities in Ebonyi, Ogun, Delta, Enugu, Plateau, Katsina, Gombe, Osun, and Abia States were compiled.

To ensure consistency in data collection across study sites, all enumerators underwent standardized training on product identification, nutrient information extraction, and image capture procedures. Detailed nutritional information from back-of-pack labels was documented using a smartphone-based data collection system. Enumerators were also required to capture clear photographs of all sides of each product package. As a quality assurance measure, the label images were reviewed to validate the recorded data, ensuring the accuracy and completeness of the information entered into the FLAIR database.

To avoid duplication, products recorded from multiple locations were cross-checked and consolidated. Different package sizes of the same product were treated as duplicates and excluded. Only products from the same brand with distinct organoleptic attributes or compositional constituents were captured.

### 2.3 Data categorization

#### 2.3.1 Food categories

This study followed the International Network for Food and Obesity/non-communicable Diseases Research, Monitoring and Action Support (INFORMAS) protocol to categorize the pre-packaged products into several groups: confectionary, bread and bakery products, cereal, and cereal products, dairy, edible oils and oil emulsions, fruit and vegetables, sauces and spreads, snack foods, processed fish; special foods, meat and meat products, non-alcoholic beverages, and alcohol (38). An additional category of processed tuber products was included because tuber-based starchy staples, such as yam flour and cassava derivatives, constitute a prominent component of the Nigerian diet and food supply (39). Owing to their distinct characteristics, these products could not be appropriately classified within the existing INFORMAS food categories.

#### 2.3.2 Nutrient profile model

The WHO developed the African Nutrient Profile Model, which is designed to support member states in this region in controlling the obesogenic food environment by restricting the marketing of unhealthy foods to children (25). The NPM thresholds for this study were derived from the African Region Nutrient Profile Model, which established acceptable limits for major nutrients of concern across various product categories (25). Although the WHO nutrient profiling was based on the food grouping system outlined in the Codex General Standard for Food Additives (GSFA) online classification, the results were reported using the INFORMAS food categories, which offer a more concise set of groupings that can be more easily summarized in tables for computation and reporting. Thus, products classified according to the INFORMAS food categories were mapped to the corresponding categories within the African Nutrient Profile Model, where marketing thresholds were available. These products were subsequently assessed to determine the extent to which their nutrient contents exceeded the specified thresholds for nutrients of concern. The relevant nutrient thresholds and corresponding product categories are presented in Table 1.

**Table 1:** The nutrient profile model thresholds across food categories.

Variable	Saturated fat	Total fat	Total sugar	Added sugar	Sodium	Energy
Confectionery	NTP^a^	8.0	6.0	NTP^a^	NTP^a^	230
Bread and bakery products	NTP^a^	8.0	6.0	NTP^a^	0.25	230
Cereal and cereal products	NTP^a^	12	9.0	NTP^a^	0.35	NTP^a^
Dairy	NTP^a^	4.0	NTP^a^	0	NTP^a^	NTP^a^
Edible oils and oil emulsions	35	NTP	NTP^a^	0	0.10	NTP^a^
Fruit and vegetables	NTP^a^	5.0	NTP^a^	0.0	0.4	NTP^a^
Sauces and spreads	NTP^a^	8.0	NTP^a^	0	0.3	NTP^a^
Snack foods	NTP^a^	8.0	NTP^a^	0	0.25	230
Processed fish	3.0	8.0	NTP^a^	NTP^a^	0.4	NTP^a^
Special foods	NTP^a^	NTP^a^	6.0	0.0	0.1	NTP^a^
Meat and meat products	3.0	8.0	NTP^a^	NTP^a^	0.4	NTP^a^
Non-alcoholic beverages	NTP^a^	NTP^a^	0.0	NTP^a^	0.1	NTP^a^

^a^NTP, No threshold provided.

### 2.4 Data analysis

The study collated relevant data from the FLAIR database for analysis. Undeclared nutrients for a product were treated as missing data to avoid influencing the computed mean. Nutrients declared as negligible or zero were included in the analysis. Nutrient content was expressed per 100 g or 100 ml, as appropriate. Energy was reported in kcal per 100 g or 100 ml, while sodium and cholesterol were reported in mg per 100 g or 100 ml.

Descriptive analysis was used to summarize the nutritional quality of pre-packaged foods by food category. Nutrient disclosure was reported as the frequency and proportion of products with non-missing label information for each nutrient. Nutrient composition was summarized using mean values with 95% confidence intervals, as well as median and interquartile range to account for skewed distributions. To support comparison across nutrients measured on different scales, median nutrient values were also standardized within each nutrient and displayed as a heatmap across food categories.

Nutrient profile model assessment was conducted by estimating the proportion of products that exceeded the relevant threshold for each nutrient within each food category, as well as the proportion that exceeded at least one threshold. Results were presented in tables and heatmaps for clarity. All summary statistics were conducted using IBM SPSS Statistics (version 28, IBM Corp., Armonk, NY, USA), while R software (version 4.6, R Foundation for Statistical Computing, Vienna, Austria) was used for heat map visualizations.

## 3 Results

A total of 883 food products were retrieved, analyzed and distributed disproportionately across the INFORMAS food categories: alcoholic beverages (167, 18.9%), including beer, wine, and spirits; fruits and vegetables (127, 14.4%); non-alcoholic beverages (103, 11.7%), including carbonated soft drinks and fruit drinks, bread and bakery products (96, 10.9%); confectionery (92, 10.4%); dairy products (91, 10.3%), including milk, yogurt, and cheese products; cereal and cereal products (73, 8.3%), including breakfast cereals, instant noodles, and pasta products; snack foods (32, 3.6%); processed tuber products (24, 2.7%), such as cassava- and yam-based products; edible oils and oil emulsions (22, 2.5%); sauces and spreads (21, 2.4%); special foods (20, 2.3%); processed fish (12, 1.4%); and meat and meat products (3, 0.3%).

### 3.1 Frequency of nutrient content declaration in pre-packaged foods

**Figure 1:**
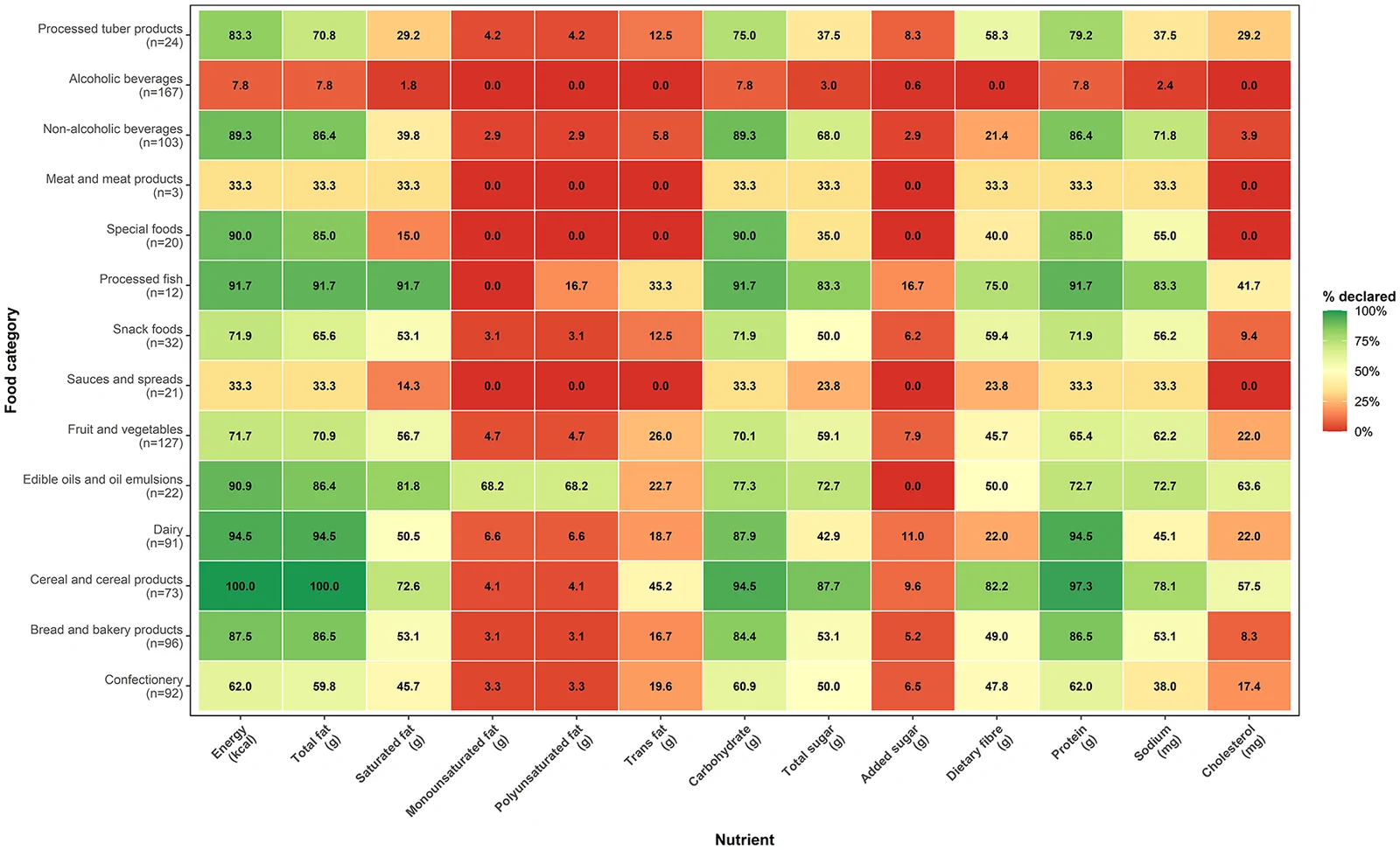
Percentage of pre-packaged food products with nutrient information disclosed by food category. All nutrient values are expressed per 100 g or 100 mL, as appropriate.

Figure 1 presents the frequency of nutrient content declaration among pre-packaged foods. Overall, with the exception of products in the sauces and spreads, meat and meat products, and alcoholic beverages categories, the majority of products (>60.9%) reported information on energy, total fat, protein, and carbohydrate content.

Information on nutrient content declaration across food categories showed that energy content was declared in the majority of products within the cereal and cereal products (100.0%), dairy products (94.5%), processed fish (91.7%), special foods (90.0%), non-alcoholic beverages (89.3%), and bread and bakery (87.5%) products. Less than one-tenth of alcohol (7.8%) reported their energy content, while energy content disclosure was observed in one-third of sauces and spreads, and meat and meat products.

A significant proportion of pre-packaged products reported their fat and saturated fat information: cereal and cereal products (fat: 100%; saturated fat: 72.6%), dairy products (fat: 94.5%; saturated fat: 50.5%), processed fish (fat: 91.7%; saturated fat: 91.7%), bread and bakery (fat: 86.5%; saturated fat: 53.1%), edible oil and oil emulsions (fat: 86.4%; saturated fat: 81.8%), and fruit and vegetables (fat: 70.9%; saturated fat: 56.7%). The declaration of other fat subsets, such as cholesterol, monounsaturated fat, polyunsaturated fat, and trans-fat, was generally less common, except for the cholesterol content of cereal and cereal products (57.5%), edible oils and oil emulsions (63.6%), and processed fish (41.7%).

High and comparable levels of reporting for both carbohydrate and sugar were observed among cereal and cereal products (carbohydrate: 94.5%, sugar: 87.7%), processed fish (carbohydrate: 91.7%, sugar: 83.3%), and non-alcoholic beverages (carbohydrates: 89.3%; sugar: 68.0%). However, substantial variation in the reporting of carbohydrate and sugar content was observed among bread and bakery products (carbohydrate: 84.4%, sugar: 53.1%), dairy products (carbohydrate: 87.9%, sugar: 42.9%), and special foods (carbohydrate: 90.0%, sugar: 35.0%). Added sugar was largely unreported in over 90% of all food categories. Cereal and cereal products (82.2%) had the highest disclosure of fiber content, followed by processed fish (75.0%) and snack foods (59.4%). Protein content was reported in the majority of the following pre-packaged foods: bread and bakery products (86.5 %), cereals and cereal products (97.3 %), dairy (94.5 %), processed fish (91.7 %), special foods (85.0%), and non-alcoholic beverages (86.4 %).

### 3.2 Nutrient composition of pre-packaged foods across food categories

**Figure 2:**
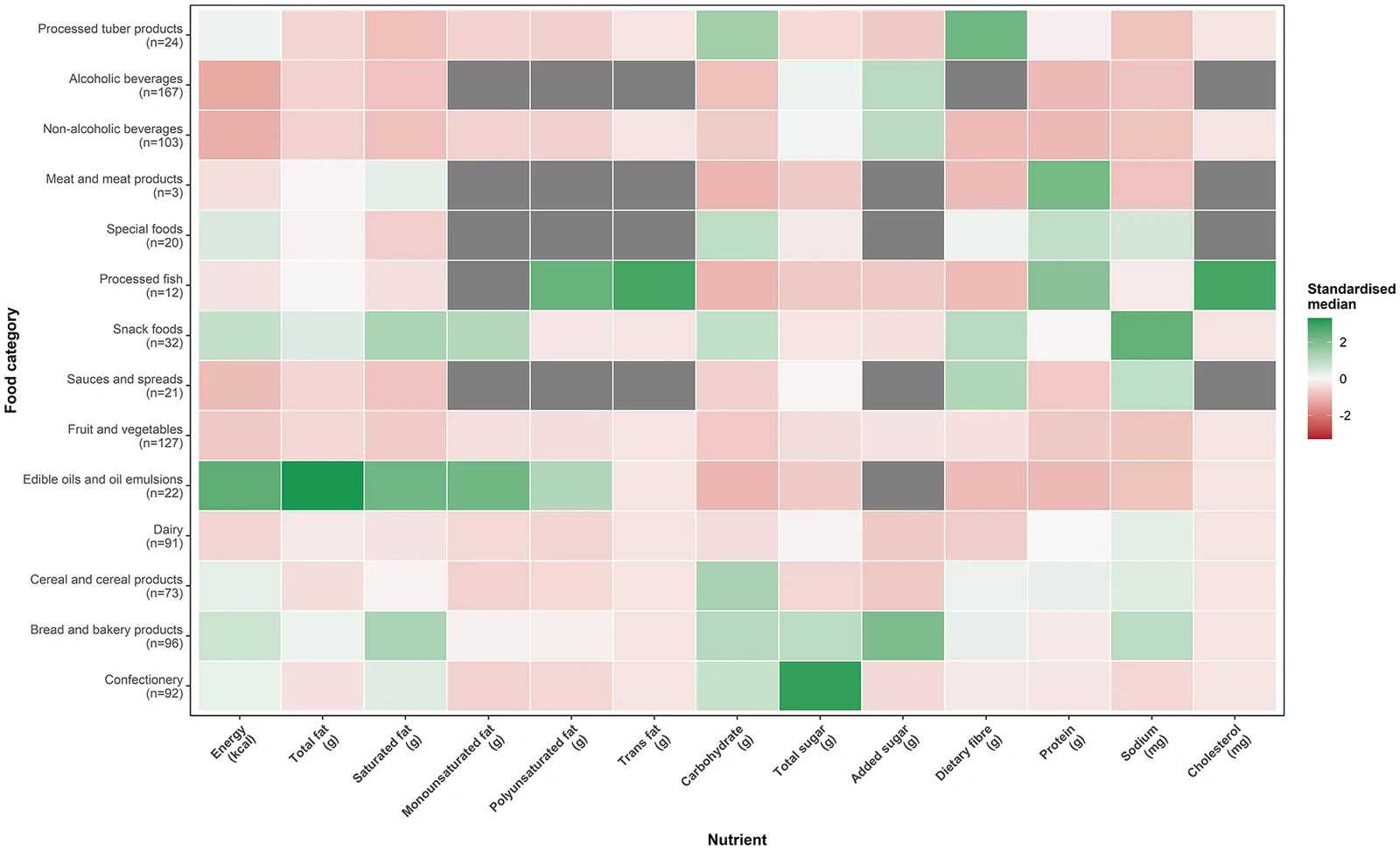
Standardized median nutrient content of pre-packaged food products across food categories. Cell values represent median nutrient content by food category, standardized within each nutrient using z-scores for visual comparison across nutrients measured on different scales. Positive values indicate above-average medians and negative values indicate below-average medians across food categories. All nutrient values are expressed per 100 g or 100 mL, as appropriate.

Information on the mean nutrient content of pre-packaged food products by food category, together with the corresponding confidence intervals, is presented in Tables 2, 3. These findings were complemented by a visual illustration of the distribution of standardized median nutrient contents across food categories, with median values standardized within each nutrient to facilitate comparison across categories (Figure 2). Detailed median values and interquartile ranges are provided in the Supplementary Tables S1, S2.

**Table 2:** Mean macronutrient, cholesterol and sodium content of pre-packaged foods by food categories.

Variable	Energy (kcal/100 g)		Fat (g/100 g)		Carbohydrate (g/100 g)		Protein (g/100 g)		Cholesterol (mg/100 g)		Sodium (mg/100 g)	
	Mean	CI	Mean	CI	Mean	CI	Mean	CI	Mean	CI	Mean	CI
Confectionery	338.55	295.83, 381.60	12.75	9.22, 16.39	50.77	43.71, 58.20	6.47	4.30, 9.98	1.75	0.22, 3.40	84.56	46.94, 128.91
Bread and bakery products	408.70	377.90, 436.52	18.36	16.34, 20.17	56.76	52.32, 60.90	5.85	5.16, 6.60	4.73	0.00, 11.57	321.45	249.58, 395.39
Cereal and cereal products	383.56	356.96, 408.68	8.40	6.78, 10.07	69.83	66.97, 72.72	9.93	8.99, 11.01	1.32	0.00, 3.79	686.55	493.47, 874.52
Dairy	266.83	228.53, 305.75	14.33	12.10, 16.82	25.17	20.87, 29.57	11.01	9.08, 12.98	6.92	2.06, 13.25	157.10	114.17, 201.07
Edible oils and oil emulsions	722.88	553.56, 854.49	84.54	68.64, 98.50	0.00	0.00, 0.00	0.01	0.00, 0.05	0.00	0.00, 0.00	0.03	0.00, 0.11
Fruit and vegetables	233.13	173.06, 290.19	15.93	10.63, 21.53	17.78	13.43, 22.16	4.96	3.36, 6.90	3.68	0.35, 9.38	835.33	118.00, 1,986.48
Sauces and spreads	99.46	83.79, 110.90	3.21	0.92, 6.13	11.79	7.25, 16.22	3.99	1.07, 6.76			213.43	145.50, 291.43
Snack foods	679.76	465.99, 1106.61	49.59	25.25, 109.34	54.51	48.74, 59.01	8.86	7.36, 10.98	0.00	0.00, 0.00	6,448.78	247.60, 20,873.80
Processed fish	270.46	150.56, 469.84	20.92	7.67, 42.57	0.68	0.03, 1.65	19.24	15.10, 23.24	54.17	16.50, 104.47	142.81	30.77, 274.78
Special foods	372.89	304.23, 439.99	12.90	9.62, 16.47	51.78	41.30, 60.35	14.22	10.46, 19.16			136.54	94.93, 176.68
Meat and meat products	226.00	226.00, 226.00	14.00	14.00, 14.00	0.00	0.00, 0.00	25.00	25.00, 25.00			0.64	0.64, 0.64
Non-alcoholic beverages	46.92	39.24, 56.48	0.42	0.03, 1.26	10.60	9.43, 11.73	0.20	0.10, 0.34	0.00	0.00, 0.00	4.82	2.52, 7.91
Alcohol	50.99	20.52, 87.27	0.38	0.03, 1.20	11.74	4.70, 19.97	0.16	0.03, 0.35			3.78	0.01, 10.00
Processed tuber products	315.72	254.4, 361.92	2.43	1.05, 4.09	73.48	64.32, 79.92	6.70	4.61, 8.67	0.00	0.00, 0.00	88.04	9.46, 192.48

**Table 3:** Fat and carbohydrate subcomponent contents of pre-packaged food products by food categories.

Variable	Saturated fat (g/100 g)		Mono-unsaturated fat (g/100 g)		Poly-unsaturated fat (g/100 g)		Transfat (g/100 g)		Sugar (g/100 g)		Added sugar (g/100 g)		Dietary fiber (g/100 g)	
	Mean	CI	Mean	CI	Mean	CI	Mean	CI	Mean	CI	Mean	CI	Mean	CI
Confectionery	8.81	6.45, 11.34	4.87	0.00, 14.60	0.93	0.50,1.70	0.03	0.00, 0.09	39.96	31.93, 48.11	3.70	0.76, 7.71	1.56	1.18, 1.96
Bread and bakery products	8.87	7.42, 10.39	5.87	5.00, 6.60	5.07	3.00, 8.30	0.13	0.00, 0.43	21.71	18.12, 25.23	19.72	11.34, 26.19	3.02	2.27, 3.87
Cereal and cereal products	4.31	3.29, 5.42	0.00	0.00, 0.00	0.93	0.89, 1.00	0.00	0.00, 0.00	6.27	4.35, 8.30	0.00	0.00, 0.00	3.23	2.52, 4.02
Dairy	6.67	4.49, 9.02	1.50	0.83, 2.20	11.87	0.26, 26.33	1.08	0.01, 2.76	11.87	8.10, 16.10	7.05	0.00, 16.39	0.46	0.25, 0.64
Edible oils and oil emulsions	27.23	15.36, 40.98	37.81	28.78, 47.43	28.50	16.94, 40.46	0.20	0.00, 0.67	0.00	0.00, 0.00	0.00		0.00	0.00, 0.00
Fruit and vegetables	2.96	1.95, 4.06	4.35	0.66, 8.63	2.75	0.17, 5.50	0.65	0.38, 1.40	13.72	8.90, 18.23	6.18	1.41, 14.27	2.92	1.94, 4.00
Sauces and spreads	0.33	0.20, 0.50	0.00		0.00		0.00		9.10	7.50, 11.00	0.00		5.21	4.20, 6.70
Snack foods	8.45	6.13, 10.75	21.70	21.70, 21.70	2.80	2.80, 2.80	0.00	0.00, 0.00	9.34	4.68, 15.63	2.65	1.30, 4.00	11.36	3.94, 21.09
Processed fish	2.48	1.54, 3.59	0.00		25.20	8.40, 42.00	1.40	0.00, 5.50	0.10	0.00, 0.30	0.00	0.00, 0.00	1.56	0.00, 4.49
Special foods	1.17	0.90, 1.50	0.00		0.00		0.00		12.32	4.70, 21.11	0.00		2.70	1.32, 4.23
Meat and meat products	5.50	5.50, 5.50	0.00		0.00		0.00		0.00	0.00, 0.00	0.00		0.00	0.00, 0.00
Non-alcoholic beverages (powdered and liquid)	0.45	0.01, 151	0.00	0.00, 0.00	0.00	0.00, 0.00	0.00	0.00, 0.00	9.95	8.76, 11.20	8.60	0.00, 13.00	0.07	0.00, 0.15
Alcohol	0.97	0.00, 2.70	0.00		0.00		0.00		15.92	3.39, 29.36	13.00	13.00, 13.00	0.00	
Processed tuber products	0.17	0.00, 0.42	0.00	0.00, 0.00	0.00	0.00, 0.00	0.00	0.00, 0.00	2.33	0.87, 3.78	0.00	0.00, 0.00	5.11	3.79, 6.65

Edible oils and oil emulsions (722.88 kcal; CI = 553.56, 854.49), Snack foods (679.76 kcal; CI= 465.99, 1,106.61), bread and bakery products (408.70 kcal; CI= 377.90, 436.51), and cereal/cereal products (383.56 kcal; CI=356.96, 408.68) had higher energy content than other product categories. While non-alcoholic beverages, and sauces and spreads recorded the lowest energy content at (46.91 kcal; CI= 39.24, 56.48) and (99.46 kcal; CI= 83.79, 110.99), respectively. This pattern was also reflected in the standardized median heatmap, where edible oils and oil emulsions, snack foods, special foods, and bread and bakery products displayed positive standardized median energy values, indicating relatively higher median energy contents compared with other food categories.

The highest fat content was reported in edible oils and oil emulsions (85.54 g; CI = 68.64, 98.50), followed by snack foods (49.59 g; CI = 25.25, 109.34), fruit and vegetables (15.93 g; CI = 10.63, 21.53) and bread and bakery products (18.36 g; CI = 16.34, 20.17). However, while this aligned closely with the median, snack foods and fruit and vegetable products exhibited substantially lower median fat content than their means. Alcohol (0.4 g; CI = 0.03, 1.20) and non-alcoholic beverages (0.4 g, CI = 0.03, 1.26) had the lowest fat content.

Processed fish products had the highest cholesterol content (54.17 mg; CI = 16.50, 104.47), substantially exceeding that of all other food categories. Dairy products (6.92 mg; CI = 2.06, 13.25) and bread and bakery products (4.73 mg; CI = 0.00, 11.57) ranked second and third, respectively. However, the relatively higher mean cholesterol contents observed in dairy and bread and bakery products were not reflected in the standardized median values, which remained comparatively low. Notably, edible oils and oil emulsions, snack foods, non-alcoholic beverages, and processed tuber products reported negligible values (0.0 mg, CI = 0.00, 0.00).

Processed tubers (73.48 g, CI = 64.32, 79.92), cereals and cereal products (69.83 g, CI = 66.97, 72.72), bread and bakery products (56.76 g, CI = 52.32, 60.90), special food (51.78 g, CI = 41.30, 60.35), snack foods (54.51 g, CI = 48.74, 59.01) and confectionery (50.77 g, CI = 43.71, 58.20) contained high carbohydrate amounts. Similarly, these patterns are clearly reflected in the standardized median values. Meat and meat products, processed fish, special foods and dairy contained higher protein levels ranging from 11 to 25.00 g, with slightly comparable patterns reported across standardized median levels. On the other hand, edible oils and oil emulsions, and non-alcoholic beverages contain negligible amounts of protein: 0.01 g (CI = 0.00, 0.05) and 0.20 g (CI = 0.10, 0.34).

The highest sodium content was found in snack foods (6,448.78mg, CI = 247.60, 20,873.80), fruit and vegetables (835.33 mg, CI= 118.00, 1,986.48), and bread and bakery products (321.45, CI = 249.58, 395.39) while edible oils and oil emulsions (0.03 mg, CI = 0.00, 0.11) and meat and meat products (40.64 mg, CI = 0.64, 0.64) contained insignificant amounts. In contrast, the median (interquartile range) sodium values of snack foods, 377.60 mg (419.18) and fruits and vegetables, 5 mg (239.86), were reflected by substantially lower standardized median values relative to the means, indicating a right-skewed distribution.

Compared to other product categories, edible oils and oil emulsions had the highest amounts of saturated fat (27 g; CI = 15.36, 40.98), monounsaturated fat (37.81 g; CI=28.78, 47.43), and polyunsaturated fat (28.50 g; CI = 16.94, 40.46). Alcoholic, non-alcoholic beverages, sauces and spreads, and processed tubers contained insignificant amounts of saturated fats (< 0.50g), monounsaturated fats ( ≤ 0.2g), and polyunsaturated fats (0.0g). Dairy products exhibited relatively high polyunsaturated fat content (11.87 g; CI = 0.26, 26.33). In addition, dairy products and processed fish contained mean trans-fat levels ranging from 1.10 to 1.40 g per 100 g. The standardized median values generally mirrored the mean values of these products, with the highest or lowest average nutrient contents of individual fat subcomponents. An exception was observed for dairy products, where the median levels of polyunsaturated and trans fats were substantially lower than their corresponding means, indicating a positively skewed distribution.

Except for confectionery products, which had the highest sugar content (39.96 g, CI = 31.93, 48.11), bread and bakery products (sugar: 21.71 g, CI = 18.12, 25.23; added Sugar: 19.72, CI = 11.34, 26.19) and alcohol (sugar: 15.92 g, CI = 3.39, 29.36; added sugar: 13.00 g, CI = 13.00, 13.00) had very high amounts of sugar and added sugar compared to other food categories. Snack foods (11.36 g, CI = 3.94, 21.09), sauces and spreads (5.21 g, CI = 4.20, 6.70), and processed tubers (5.11 g, CI = 3.79, 6.65) contained more dietary fiber than other products. Snack foods exhibited substantially lower median values relative to their means, in contrast to the other highlighted product categories, whose standardized median values were largely comparable to their mean values.

### 3.3 Proportion of prepackaged foods exceeding thresholds for key nutrients of concern

**Figure 3:**
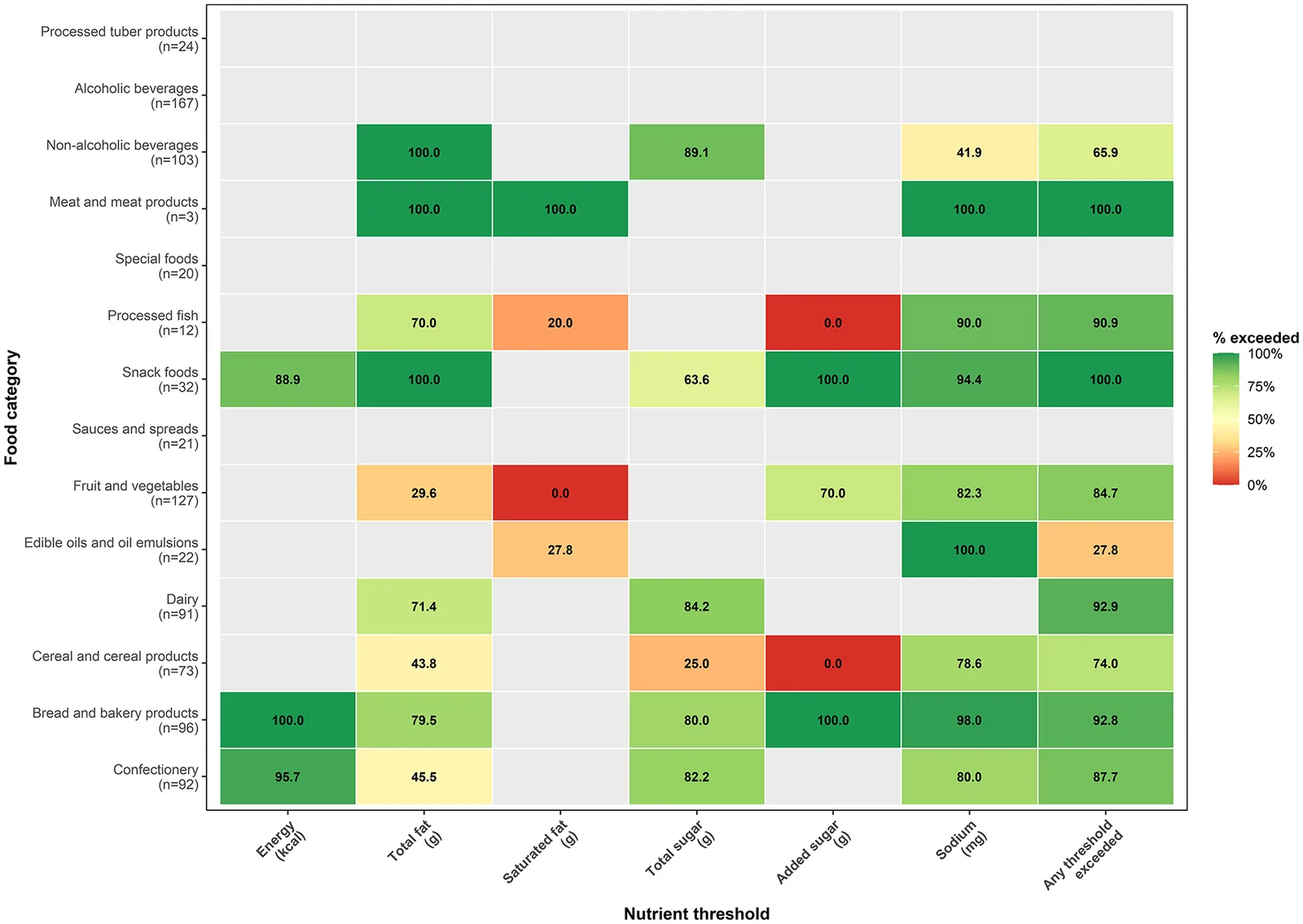
Percentage of pre-packaged food products exceeding nutrient profile model thresholds by food category and nutrient. Cell values represent the percentage of products exceeding the nutrient-specific threshold within each food category, displayed to one decimal place. The final column indicates the percentage of products exceeding at least one threshold among those with threshold data available. All nutrient values are expressed per 100 g or 100 mL, as appropriate.

The results for the threshold levels exceeded by pre-packaged foods are summarized in Figure 3. Foods within the alcoholic beverages, special foods, sauces and spreads, and processed tuber products categories were either not covered by the nutrient profile model thresholds or lacked disclosure of the nutrients for which thresholds had been established.

All snack foods, meat and meat products, and non-alcoholic beverages exceeded the recommended threshold for total fat. Furthermore, all meat and meat products (100.0%) exceeded the saturated fat threshold, followed by edible oils and oil emulsions, with 27.8% exceeding the threshold. All bread and bakery products (100.0%) exceeded the energy threshold, alongside the majority of confectionery products (95.7%) and snack foods (88.9%). Sugar thresholds were exceeded by most products within the confectionery (82.2%), bread and bakery products (80.0%), dairy (84.2%), non-alcoholic beverages (89.1%), and snack food (63.6%) categories. In addition, all bread and bakery products (100.0%) and snack foods (100.0%) exceeded the added sugar threshold. At least 9 in 10 products in the processed fish (90.0%), snack foods (94.4%), bread and bakery products (98.0%), edible oils and oil emulsions (100.0%), and meat and meat products (100.0%) categories exceeded the sodium threshold.

Overall, all meat and meat products (100.0%) and snack foods (100.0%) exceeded at least one nutrient threshold. This was followed by high proportions of bread and bakery products (92.9%), dairy products (92.8%), confectionery products (87.8%), and fruit and vegetable products (84.7%) exceeding at least one nutrient threshold.

## 4 Discussion

The widespread presence of pre-packaged foods across all food categories observed in this study reflects broader changes in Nigeria's food environment. Urbanization, globalization, affordability, and extensive marketing have increased demand for convenient, shelf-stable food products, contributing to their growing consumption and integration into everyday diets (1–5). These shifts underscore the need for closer scrutiny of the nutritional quality of pre-packaged foods to inform regulatory actions, evaluate industry labeling practices, and better understand the potential health risks associated with ongoing dietary and nutrition transitions.

However, the lower representation of categories such as processed tuber products, sauces, fish, and meat products, relative to alcoholic and non-alcoholic beverages, bakery products, confectionery, dairy products, and cereals, likely reflects the current structure of the Nigerian packaged food environment. These less-represented categories are still predominantly distributed through traditional retail channels and are commonly sold in fresh, minimally processed, or non-packaged forms rather than as branded, pre-packaged products.

The observed gaps in the nutrients in this study align with previous studies, which revealed that macronutrients often referred to as the “big four” or “priority” nutrients (energy, fat, carbohydrate and protein) are typically well-declared, with a decline in disclosure recorded when the scope is expanded to include cholesterol, sodium, and sub-components of fats and carbohydrate (40–42). This is further supported by the national regulatory transition from a longstanding voluntary nutrient-declaration guideline to a mandatory requirement for key nutrients in 2022 (16, 17). Thus, while changes are expected to take effect over time, the exceptions applied to products with small surface area and single ingredients indicate that food categories such as sauces and spreads with overall low declarations of all nutrients may not improve. Alcoholic beverages, another product category with predominantly low nutrient disclosure, have historically been exempted from nutritional labeling policies or regulated by a different agency (43, 44).

The substantial variability in the nutrient composition of pre-packaged foods in Nigeria, reflected in wide confidence intervals and differences between mean and median values within and across food categories, suggests considerable heterogeneity in product formulation and composition, including the presence of outlier values that may influence overall estimates. This variation is consistent with reported trends in the evolving Nigerian pre-packaged food supply described in previous studies (5, 29). This observed pattern of poor nutritional quality in pre-packaged foods, as reflected in elevated levels of these nutrients of concern, closely aligns with global trends. Specifically, the substantial energy content in the edible oils, oil emulsions, and snack foods category is comparable to reports from Guatemala and China, where pre-packaged foods had slightly higher energy levels (42). On the other hand, the high sodium content observed in this study corroborates findings from Guatemala and Saudi Arabia (45, 46), with even higher levels in China (42). This is not surprising, as national reports have shown that prepackaged products account for almost half of overall salt intake in Nigeria (32). Furthermore, food categories such as processed meats, snack foods, and sauces (particularly spices and condiments), which exhibited notably higher sodium levels compared to others, align with findings from a systematic review by Webster et al. (47).

High sugar content, particularly in non-alcoholic beverages, confectionery, and bakery products, is comparable to studies from Mexico and Guatemala (47, 48), where these food categories were highly implicated. Also, the growing consumption of non-alcoholic beverages, confectionery, and bakery products, or their contribution to carbohydrate intake, reported amongst the Nigerian population, including children (29, 32), may be attributed to the surge in Obesity and related complications in Nigeria (49). Furthermore, the low fiber content across most food categories aligns with reports from Thailand and China, where over 80% of pre-packaged foods were below the recommended fiber levels (47).

The predominance of sub-fat components, particularly saturated and monounsaturated fats, among pre-packaged foods within the edible oil category, as well as in other oil-based products such as snack foods, confectionery, and bakery items (50), has also been documented in previous studies (51, 52). The high levels of these unfavorable fatty acids warrant public health attention toward moderation, given the hyperpalatability of fat-dense foods (53). On the other hand, processed fish products stood out for their more health-beneficial fatty acid profile, characterized by a higher proportion of polyunsaturated fats. Furthermore, in line with study findings, another study has confirmed the presence of trans-fat in Nigerian pre-packaged foods (54), notably in a few food categories, such as edible oils and bakery (fried) products (54, 55). As the mean trans-fat content across product categories falls below the mandatory 2 g limit (56), there is a need to strengthen reformulation efforts toward the progressive elimination of trans-fat.

A considerable proportion of products exceeded established marketing thresholds for critical nutrients across various food categories. These findings highlight the potential for widespread exposure to excessive levels of saturated fat, total fat, sugar, added sugar, sodium, and energy, raising concerns about public health implications in Nigeria. The prevalence of these exceedances suggests an increased risk of non-communicable diseases (NCDs) as extensive pooled evidence has an established link between pre-packaged foods high in free sugars, especially those from sugar-sweetened beverages (57, 58), sodium (59, 60), and saturated fats (61) with obesity, hypertension, type 2 diabetes, cardiovascular disease, and other metabolic and non-communicable diseases.

Specifically, while pre-packaged foods within the meat, fish, and edible oils categories frequently exceeded saturated fat thresholds, snack foods, confectionery, and bakery products accounted for the largest proportion of items exceeding total fat and sugar limits. Notably, these product categories correspond to pre-packaged foods that are widely and regularly consumed nationally, such as vegetable oils, sugar and sugary foods and beverages like soft drinks, wheat flour products, and salty processed foods (31, 32). This underscores the need for stricter marketing regulations to curb excessive consumption and mitigate potential adverse health outcomes, particularly for children (62).

Furthermore, our analysis indicated that a significant percentage of pre-packaged foods exceeded the recommended threshold for sodium, ranging from (93.6%−100%) in sauces and spreads, processed fish, and bread/bakery products. Therefore, following the global call for a 20% reduction in sodium intake (63), Nigeria has recently strengthened its commitment to sodium-reduction plans by introducing national guidelines and regulations for pre-packaged foods (64, 65). This progress is expected to translate into achieving a score of two (2) on the global benchmarks, similar to that attained by other West African countries such as Guinea, Mauritania, and Senegal (66).

Placing this study report within the context of global nutrient profiling systems, our study, which reported substantial levels of thresholds exceeded by pre-packaged food products, aligns with findings from several studies which employed the regional or national nutrient profiling model, which showed that most pre-packaged foods available in Chile, Canada, Guatemala and Mexico exceeded at least one negative nutrient (14, 42, 48). Similarly, another study in Switzerland demonstrated that more than half (>58%) of pre-packaged foods aimed at children received a Nutri-Score of D or E, indicating less healthy nutritional profiles (67).

Finally, improving food labeling practices through more comprehensive and accurate disclosure of key nutrients would enhance the transparency and reliability of nutrient profile assessments. Regulatory efforts should not only strengthen enforcement of existing mandatory nutrient disclosure requirements but also establish and monitor clear product reformulation targets to reduce nutrients of concern. In addition, policies that increase the affordability and accessibility of healthier food options, while discouraging the consumption of less healthy products through marketing restrictions and fiscal measures such as taxation, may be particularly important in low-resource settings. Collectively, these strategies could help create a healthier food environment and support efforts to curb the growing burden of diet-related non-communicable diseases in Nigeria.

## 5 Conclusion

Nutrition declaration varied across both food categories and individual nutrients. While spices and sauces, meat and meat products, and alcoholic beverages showed the lowest levels of declaration amongst food categories, sub-fat components, added sugars, and cholesterol were the least frequently reported. This underscores the need for increased transparency to empower consumers' informed decisions.

Edible oil had the highest levels of fat and all fat-related components, while sodium and energy were notably high in snack foods. Elevated carbohydrate and sugar levels were attributed to bread/bakery products and snack foods, with processed fish being a dominant source of protein and cholesterol. The thresholds for total fat, total and added sugars, sodium, and energy were exceeded in a majority of the affected product categories.

In parallel, these findings are consistent with regional and international settings, where pre-packaged foods are frequently characterized by high levels of calories, sugar, saturated fat, and sodium, underscoring the need for collective global action to improve their nutrient composition. At the national level, there is a need for policymakers and stakeholders to establish, where lacking, and strictly enforce regulations on labeling and nutrient composition, including mandatory nutrient declarations, national limits for harmful nutrients, and product reformulation targets, while also creating a supportive environment to facilitate industry compliance. Furthermore, efforts to discourage the consumption of less healthy products through marketing restrictions and fiscal policies, such as taxation, should be complemented by subsidies and other interventions that improve the affordability and accessibility of healthier foods, particularly for vulnerable populations and low-income households.

## 6 Limitations of study

This study is the first to comprehensively assess the nutritional quality of pre-packaged foods in Nigeria, selected from diverse geopolitical zones. The key nutrient of concern for various food categories was reported in this study, and these findings are comparable to those reported in the literature.

However, the following limitations should be acknowledged. This study relied solely on information provided on food product labels, without any chemical evaluation, implying that any potentially inaccurate information on the labels may have been captured as part of the study's findings. Furthermore, the absence of direct verification by manufacturers in this supermarket/store audit study increases the likelihood of identifying adulterated products.

Finally, substantial heterogeneity in nutrient content was observed across several product categories and undeclared nutrients were counted as unavailable information in this study; therefore, products that may have omitted certain nutrients due to negligible or zero amounts of specific nutrients could not be distinguished and were still tagged as “undeclared”.

## Data Availability

The raw data supporting the conclusions of this article will be made available by the authors, without undue reservation.
