# Supplementary material for: Nutritional quality of pre-packaged foods in Nigeria: findings from the NigeFE study
Source: Front Nutr. 2026 Aug 13;13:1873374. doi: 10.3389/fnut.2026.1873374 (PMC13518118; doi:10.3389/fnut.2026.1873374)
Supplement: Supplementary file 1 [file Table_1.docx]

**Suppl. Table S1: Median and Interquartile range of macronutrient, cholesterol and sodium content of pre-packaged foods by food categories**

| **Variables** | **Energy** | **Fat** | **Carbohydrate** | **Protein** | **Cholesterol** | **Sodium** |
| --- | --- | --- | --- | --- | --- | --- |
|  | **Median (IQR)** | **Median (IQR)** | **Median (IQR)** | **Median (IQR)** | **Median (IQR)** | **Median (IQR)** |
| Confectionery | 373.60 (297.70) | 6.00 (24.00) | 55.05 (37.35) | 5.70 (7.28) | 0.00 (1.00) | 38.00 (123.70) |
| Bread and bakery products | 475.30 (110.28) | 20.00 (14.00) | 63.00 (14.65) | 6.00 (3.04) | 0.00 (13.40) | 209.95 (370.00) |
| Cereal and cereal products | 382.64 (89.95) | 5.10 (15.41) | 70.00 (11.75) | 10.00 (2.93) | 0.00 (0.00) | 148.30 (1539.04) |
| Dairy | 186.40 (374.38) | 9.00 (24.43) | 18.25 (29.50) | 7.90 (20.0) | 0.01 (9.58) | 135.00 (183.00) |
| Edible oils and oil emulsions | 899.57 (72.00) | 100.00 (8.00) |  | 0.00 (0.00) | 0.00 (0.00) | 0.00 (0.00) |
| Fruit and vegetables | 140.00 (215.94) | 1.95 (19.90) | 10.00 (20.54) | 1.96 (4.90) | 0.00 (0.00) | 5.45 (239.86) |
| Sauces and spreads | 100.30 (20.60) | 1.20 (7.70) | 13.00 (11.00) | 2.20 (7.20) |  | 204.00 (220.00) |
| Snack foods | 513.60 (43.00) | 26.90 (8.30) | 57.00 (13.18) | 7.50 (2.90) |  | 377.60 (419.18) |
| Processed fish | 235.20 (130.67) | 14.30 (14.50) | 0.00 (1.00) | 22.30 (13.00) | 38.00 (83.43) | 73.12 (273.31) |
| Special foods | 426.50 (133.57) | 12.90 (11.30) | 58.75 (13.65) | 15.00 (8.65) |  | 170.00 (99.00) |
| Meat and meat products |  |  |  |  |  |  |
| Non-alcoholic beverages | 45.20 (26.85) | 0.00 (0.00) | 11.00 (6.60) | 0.00 (0.30) |  | 0.40 (5.25) |
| Alcohol | 24.50 (111.15) | 0.10 (0.10) | 5.80 (23.05) | 0.00 (0.15) |  | 2.55 (8.72) |
| Processed tuber products | 350.85 (63.11) | 1.00 (3.65) | 73.33 (15.01) | 6.67 (8.14) |  | 2.00 (166.65) |

**Suppl. Table S2: Median and Interquartile range of fat and carbohydrate subcomponents by food categories**

| **Variable** | **Saturated fat** | **Mono-unsaturated fat** | **Poly- unsaturated fat** | **Trans-fat** | **Sugar** | **Added sugar** | **Dietary fibre** |
| --- | --- | --- | --- | --- | --- | --- | --- |
|  | **Median**  **(IQR)** | **Median**  **(IQR)** | **Median**  **(IQR)** | **Median**  **(IQR)** | **Median**  **(IQR)** | **Median**  **(IQR)** | **Median**  **(IQR)** |
| Confectionery | 6.00 (14.65) | 0.00 | 0.60 | 0.00 | 49.75 (53.13) | 2.05 (6.08) | 1.45(2.44) |
| Bread and bakery products | 10.00 (7.50) | 6.00 | 3.90 | 0.00 (0.00) | 22.61 (24.70) | 20.50 (12.85) | 2.40(2.80) |
| Cereal and cereal products | 3.60 (8.04) |  | 0.89 |  | 3.00 (8.64) |  | 2.30 (2.38) |
| Dairy | 2.55 (12.88) | 1.30 (1.70) | 0.40 (34.74) | 0.00 (0.08) | 9.00 (14.30) | 0.00 (16.00) | 0.55 (1.00) |
| Edible oils and oil emulsions | 14.50 (32.93) | 36.00 (22.00) | 15.00 (49.00) | 0.00 (0.50) |  |  |  |
| Fruit and vegetables | 0.84 (3.65) | 2.75 (9.15) | 1.75 (5.75) | 0.00 (0.00) | 4.30 (14.30) | 2.95 (6.93) | 1.20 (3.65) |
| Sauces and spreads | 0.30 |  |  |  | 9.10 (3.20) |  | 4.30 (2.48) |
| Snack foods | 10.00 (9.55) |  |  |  | 5.82 (12.98) | 2.65 | 4.00 (2.20) |
| Processed fish | 2.30 (1.60) |  | 25.20 | 0.50 (4.15) | 0.00 (0.00) |  | 0.00 (2.53) |
| Special foods | 1.10 |  |  |  | 6.80 (25.00) |  | 2.25 (3.93) |
| Meat and meat products |  |  |  |  |  |  |  |
| Non-alcoholic beverages | 0.00 (0.00) |  |  |  | 11.00 (7.50) | 12.80 | 0.00 (0.00) |
| Alcohol | 0.20 |  |  |  | 12.10 (28.25) |  |  |
| Processed tuber products | 0.00 (0.33) |  |  |  | 3.33 (3.33) |  | 6.34 (4.72) |
